# Survival Outcomes in Lymph Node-Positive Merkel Cell Carcinoma (Stage III): A Comparison Between Known and Unknown Primary Tumors and Their Sun Exposure Sites

**DOI:** 10.3390/medsci14020193

**Published:** 2026-04-11

**Authors:** Ronen Brenner, Hanna T. Frumin Edri, Sabri El-Saied, Ilia Berezhnov, Anna Ievko, Keren Rouvinov, Sofiia Turaieva, Amichay Meirovitz, Tanzilya Tairov, Shlomit Fenig, Nashat Abu Yasin, Alexander Yakobson, Eyal Fenig, Abed Agbarya, Walid Shalata

**Affiliations:** 1 Oncology Institute, Edith Wolfson Medical Center, Holon 58220, Israel; 2 Faculty of Medicine, Tel Aviv University, Tel Aviv-Yafo 69978, Israel; 3 Medical School for International Health, Faculty of Health Sciences, Ben Gurion University of the Negev, Beer Sheva 84105, Israel; 4 The Legacy Heritage Cancer Center, Dr. Larry Norton Institute, Soroka Medical Center, Beer Sheva 84105, Israel; 5 Institute of Oncology, Kaplan Medical Center, Faculty of Medicine, Hebrew University, Jerusalem 91905, Israel; 6 Institute of Oncology, Davidoff Center, Rabin Medical Center, Beilinson Hospital, Petah Tikva 49414, Israel; 7 Oncology Department, Bnai Zion Medical Center, Haifa 31047, Israel

**Keywords:** Merkel cell carcinoma, unknown primary Merkel cell carcinoma, sunlight exposure, lymph node-positive Merkel cell carcinoma, survival analysis, long-term outcomes

## Abstract

**Background**: Merkel cell carcinoma (MCC) is a rare, aggressive skin cancer, with prognosis influenced by tumor location and primary status. This study evaluated clinicopathological features and survival outcomes in patients with MCC from multiple centers in Israel. **Methods**: Data on demographics, tumor characteristics, lymph node (LN) involvement, treatment, and survival were collected. Patients were stratified by primary tumor status (known vs. unknown) and tumor location (sun-exposed vs. non-sun-exposed). Disease-free survival (DFS) and overall survival (OS) were estimated using Kaplan–Meier analysis, and multivariate analyses were performed. **Results**: The cohort included 80 patients diagnosed with stage 3 (with LN involvement) MCC, of whom 52 (65%) had primary MCC with lymph node involvement, and 28 patients (35%) with unknown primary MCC. The majority were male (81.3%), with a median age of 71.2 years (range, 37–92). The median DFS and OS for the entire cohort were 24 and 32 months, respectively. Patients with unknown primary tumors had longer DFS (34 vs. 18 months; *p* = 0.0503) and OS (43 vs. 28 months; *p* = 0.0362) compared with those with known primary MCC. Non-sun-exposed tumors were associated with longer median DFS (32 vs. 18.5 months; *p* = 0.0663) and OS (41 vs. 23 months; *p* = 0.0353). Five-year survival analysis showed improved outcomes in patients with unknown primary tumors (DFS 54% vs. 35%, *p* = 0.04; OS 57% vs. 42%, *p* = 0.03) and in non-sun-exposed tumors (DFS 51% vs. 33%, *p* = 0.05; OS 57% vs. 40%, *p* = 0.04). **Conclusions**: Unknown primary status and non-sun-exposed tumor location are potentially associated with improved long-term survival in patients with MCC. These findings highlight the prognostic importance of tumor origin and anatomical site in MCC management.

## 1. Introduction

Merkel cell carcinoma (MCC) is a rare but highly aggressive neuroendocrine malignancy of the skin characterized by rapid tumor growth, early lymphatic dissemination, and a high risk of recurrence and mortality. Although it accounts for a small proportion of cutaneous malignancies, MCC is associated with a mortality rate that exceeds that of melanoma and other non-melanoma skin cancers. The disease typically affects elderly individuals and is strongly associated with ultraviolet exposure, immunosuppression, and infection with Merkel cell polyomavirus. Clinically, MCC most commonly arises in sun-exposed areas such as the head and neck or extremities and frequently presents with regional lymph node (LN) involvement at diagnosis [1,2,3]. Despite advances in diagnosis and management, the prognosis of MCC remains largely dependent on stage at presentation. Approximately 40–50% of patients develop regional LN metastases during the course of the disease, and a substantial proportion will ultimately develop distant metastatic disease. Historically, the 5-year overall survival for MCC has been reported to be approximately 60–65%, although outcomes vary considerably depending on tumor stage, patient characteristics, and treatment strategies [2,4].

An unusual clinical presentation of MCC occurs when patients present with nodal disease without an identifiable cutaneous primary tumor, a condition referred to as Merkel cell carcinoma of unknown primary (MCCUP). This phenomenon accounts for approximately 5–12% of MCC cases and is thought to result from spontaneous regression of the primary cutaneous lesion due to immune-mediated mechanisms. Interestingly, several studies have suggested that patients with MCCUP may have improved clinical outcomes compared with those with a known primary tumor, possibly reflecting a more effective anti-tumor immune response [5,6,7].

Previous retrospective analyses have reported improved overall survival and recurrence-free survival among patients with nodal MCCUP compared with stage-matched patients with a known primary tumor. For example, a cohort study evaluating patients with stage III MCC demonstrated that the presence of an occult primary tumor was associated with significantly better survival outcomes. Similarly, population-based analyses have suggested lower recurrence rates among patients with MCCUP tumors compared with those with identifiable cutaneous lesions [6,8].

However, despite these observations, the prognostic significance of LN involvement in relation to known versus MCCUP and the comparison between sun-exposed sites remains incompletely defined, particularly among patients with non-metastatic disease. Much of the currently available evidence is derived from heterogeneous cohorts that either include patients with metastatic disease or primarily focus on nodal presentations, which limits the ability to clearly delineate survival differences in localized and regionally advanced MCC, in addition to the correlation between sun-exposed sites for this diagnosis. Given the rarity of this malignancy and the limited number of large, well-characterized datasets, further retrospective analyses are needed to better understand the prognostic impact of primary tumor status. Therefore, the aim of the present retrospective study was to evaluate survival outcomes among patients with non-metastatic MCC by comparing individuals with MCCUP tumors to those with identified primary lesions and sun-exposed sites in order to clarify the prognostic implications of primary tumor detection and to improve risk stratification in this rare and aggressive disease.

## 2. Materials and Methods

### 2.1. Study Design and Patient Population

This multicenter retrospective cohort study included patients diagnosed with localized or locally advanced Merkel cell carcinoma who underwent surgical resection followed by adjuvant treatment. Patients treated between September 1985 and February 2021 were identified from three tertiary medical centers in Israel. The study was conducted in accordance with institutional ethical standards and received approval from the Institutional Review Boards of the participating centers: Rabin Medical Center, Edith Wolfson Medical Center, and Soroka Medical Center.

### 2.2. Collection of Data

Eligible patients were identified through a comprehensive search of pathology databases and oncology department registries using standardized diagnostic codes. Clinical and pathological data were retrieved from electronic medical records and entered into a predefined data collection template by trained investigators. Collected variables included demographic characteristics (age at diagnosis and sex), tumor-related factors (tumor location), and treatment information such as surgical management, administration of chemotherapy, radiotherapy protocols, and total radiation dose delivered. Patients in both treatment groups—those who received combined chemoradiation and those treated with radiotherapy alone—were managed during comparable time periods. Radiotherapy treatments were delivered according to similar institutional protocols and standard clinical practice.

**Figure 1:**
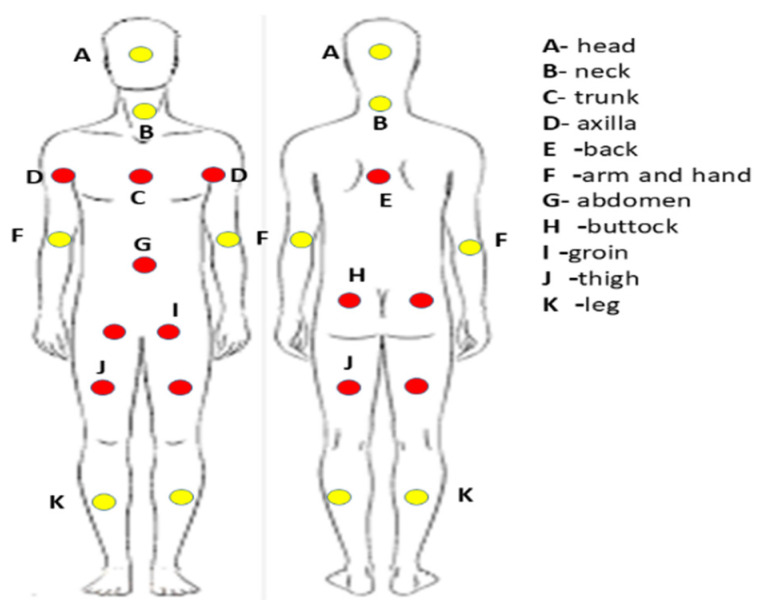
The classification of the sun-exposed areas (yellow points) and areas of the limited or no sunlight exposure (red points).

Disease stage at diagnosis was determined according to the tumor–node–metastasis (TNM) classification system defined by the American Joint Committee on Cancer staging guidelines. This staging system incorporates the extent of the primary tumor (T), regional LN involvement (N), and the presence or absence of distant metastases (M). Patients with incomplete clinical records or lacking follow-up information were excluded from the final analysis. The primary clinical outcomes evaluated were overall survival (OS) and disease-free survival (DFS). OS was defined as the interval between the date of MCC diagnosis and death from any cause or last documented follow-up. DFS was calculated from the time of diagnosis to the first documented disease recurrence, death, or last follow-up, whichever occurred first. Patients were also classified according to sun exposure status. For patients with a known primary tumor, classification was based on whether the primary lesion arose in a sun-exposed or non-sun-exposed area. For patients with MCCUP, classification was based on the anatomical location of lymph node involvement. Lymph node sites were similarly categorized into sun-exposed and non-sun-exposed regions (yellow points). Sun-exposed areas included the head and neck, arms and hands, and legs, whereas non-sun-exposed areas primarily included the axillary and groin lymph nodes, excluding the head and neck region (red points) (Figure 1). Mortality data were obtained from the national registry maintained by the Israel Ministry of Interior. The last follow-up was conducted up until May 2025.

### 2.3. Inclusion Criteria

Patients were eligible for inclusion in the study if they met the following criteria:

Age: Participants were required to be 18 years of age or older at the time of diagnosis.

Confirmed diagnosis: Histopathological confirmation of Merkel cell carcinoma with any T stage and involvement of LN (N), provided there was no evidence of distant metastatic disease (M0).

Initial treatment: Patients must have received treatment consisting of radiotherapy, chemotherapy, or a combined modality approach according to disease stage and LN involvement.

No prior treatment: Patients who had not previously received radiotherapy or systemic therapy for localized or locally advanced disease were eligible.

Treatment location and data availability: Treatment had to be administered at one of the participating medical centers, or the patient had to have complete and accessible follow-up records.

### 2.4. Exclusion Criteria

Patients were excluded from the analysis if any of the following conditions were present:

No evidence of LN involvement at diagnosis or distant metastatic disease at diagnosis.

Prior treatment with radiotherapy or systemic therapy before the initiation of the study treatment.

A final diagnosis other than Merkel cell carcinoma.

A lack of sufficient follow-up information to determine disease-free survival (DFS) or overall survival (OS).

### 2.5. Treatment Administration

Patients with Merkel cell carcinoma were treated using a multimodal approach that included radiotherapy with or without surgery, combined with systemic chemotherapy when clinically indicated. The chemotherapy regimen consisted of cisplatin administered at a dose of 20 mg/m^2^ on days 1–5 and etoposide at 100 mg/m^2^ on days 1, 3, and 5 of a 28-day cycle, for a total of four to six cycles as accepted [9]. The first two chemotherapy cycles were delivered concurrently with radiotherapy. Radiotherapy was administered to the primary tumor site and involved regional lymphatic drainage areas. The typical total radiation dose ranged between 45 and 50 Gy, delivered in 25 fractions of 1.8–2 Gy each. A sequential boost of 9–10 Gy was administered to areas of macroscopic disease when present. Treatment was delivered using either three-dimensional conformal radiotherapy (3D-CRT) or intensity-modulated radiotherapy (IMRT) with modern linear accelerators (Varian).

### 2.6. Statistical Analysis

Categorical variables were summarized using frequencies and percentages. The distribution of continuous variables was assessed visually using histograms and quantile–quantile (Q–Q) plots. Continuous variables were reported as medians with corresponding interquartile ranges (IQR). Comparisons of continuous variables between treatment groups were performed using either the independent samples *t*-test or the Mann–Whitney U test, depending on the distribution of the data. Differences in categorical variables were evaluated using the chi-square test or Fisher’s exact test, as appropriate. The reverse Kaplan–Meier method was applied to estimate the median duration of follow-up. Survival outcomes were analyzed using Kaplan–Meier methods to estimate overall survival and disease-free survival for each treatment group, and differences between curves were assessed with the log-rank test. Multivariable Cox proportional hazards regression models were used to examine the relationship between chemotherapy administration and survival outcomes while adjusting for potential confounding variables. Variables that differed significantly between groups were considered candidate confounders in the regression model. All statistical tests were two-sided, and a *p*-value < 0.05 was considered statistically significant. Statistical analyses were conducted SPSS software, version 29.0.

## 3. Results

A total of 253 patients diagnosed with MCC across multiple centers in Israel between January 1985 and December 2021 were identified in our archives. Of these, 80 patients had all the relevant information regarding our study idea, met the eligibility criteria, and were included in the analysis. Among the 80 patients included in the cohort, 28 (35%) were diagnosed with UNPMCC, while 52 (65%) had a known primary tumor. Overall, the cohort demonstrated marked male predominance (70%), which was consistent across both groups, although slightly higher in the UNPMCC subgroup (75% vs. 67.3% in MCC). The mean age at diagnosis was comparable between groups but showed a trend toward younger age in the UNPMCC group (67.8 years) compared with patients with known primary MCC (71.2 years). When stratified by sex, males in the MCC group were older at diagnosis (73.9 years) compared with males in the UNPMCC group (67.3 years), while female patients showed relatively similar age distributions between groups. All patients in the cohort presented with lymph node involvement (100%), reflecting the inclusion of stage III disease only. Accordingly, all patients underwent surgical resection followed by adjuvant therapy, with 100% receiving radiotherapy and chemotherapy as part of their treatment protocol.

A notable difference between the groups was observed in tumor location patterns. Patients with known primary MCC more frequently had tumors arising in sun-exposed areas (69.2%), whereas UNPMCC cases were predominantly associated with non-sun-exposed regions (67.9%). Conversely, only 32.1% of UNPMCC cases were linked to sun-exposed sites, compared with 30.8% of MCC cases occurring in non-sun-exposed areas (Table 1).

**Figure 2:**
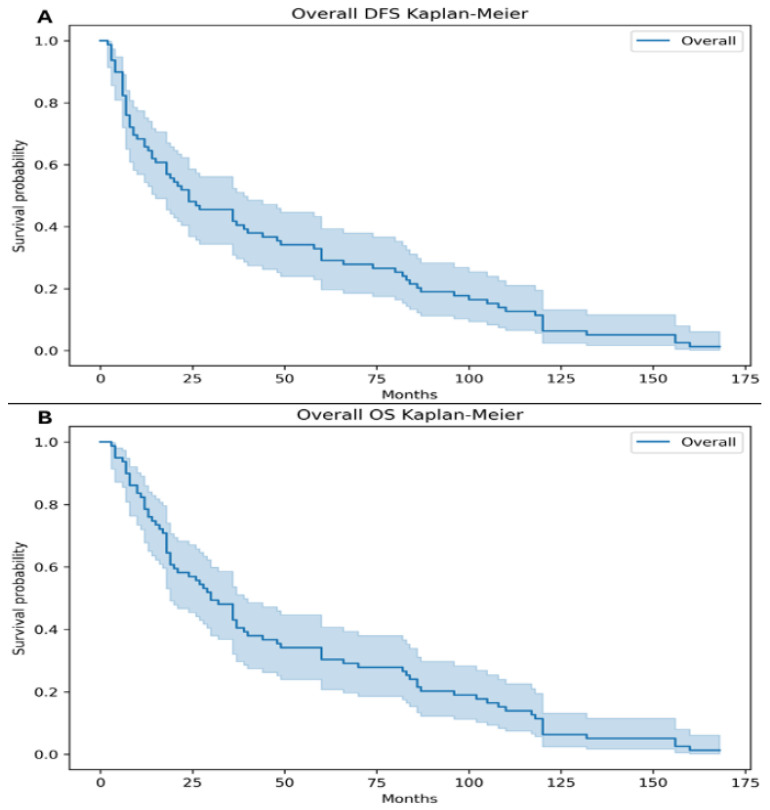
The median disease-free survival (DFS) for the entire cohort was 24 months (95% CI, 12–60) (**A**). The median overall survival (OS) was 32 months (95% CI, 14–66) (**B**).

The median DFS and OS for the entire cohort were 24 months and 32 months, respectively (Figure 2).

**Figure 3:**
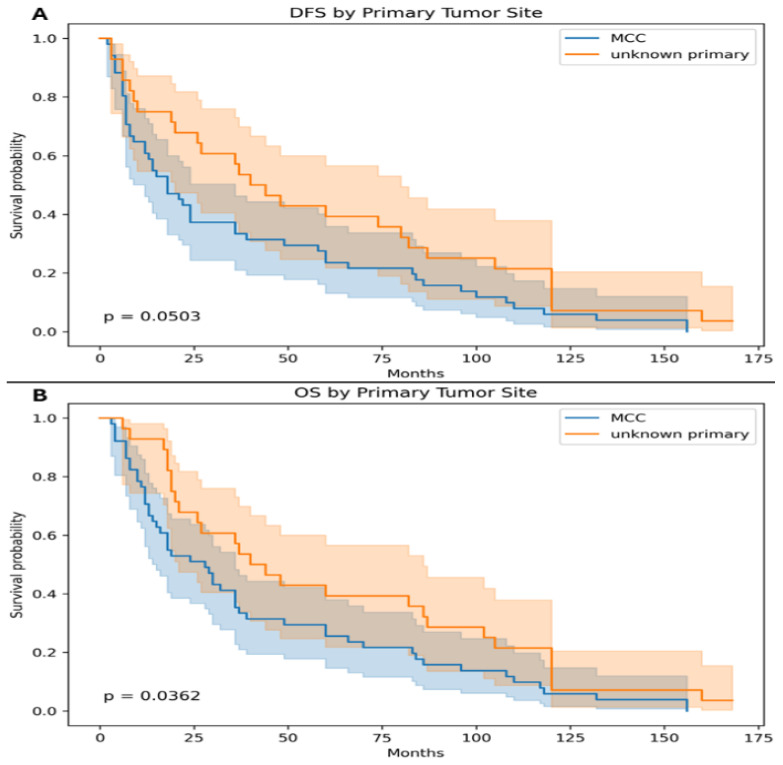
The median disease-free survival (DFS) was 34 months (95% CI, 18–120) in the unknown primary group versus 18 months (95% CI, 6–49) in patients with a known primary tumor, demonstrating a trend toward improved DFS that approached statistical significance (*p* = 0.0503) (**A**). The median overall survival (OS) was 43 months (95% CI, 19–120) for patients with unknown primary tumors compared with 28 months (95% CI, 10–70) for those with a known primary MCC. This difference was statistically significant (*p* = 0.0362) (**B**).

When comparing survival outcomes according to primary tumor status, patients with MCCUP tumors demonstrated improved survival compared with those with a known MCC primary. The median DFS was 34 months in the MCCUP group versus 18 months in patients with a known primary tumor, demonstrating a trend toward improved DFS that approached statistical significance (*p* = 0.0503). Similarly, the median OS was 43 months for patients with MCCUP tumors compared with 28 months for those with a known primary MCC. This difference was statistically significant (*p* = 0.0362), indicating that patients with MCCUP had significantly better overall survival than those with a known primary tumor (Figure 3).

**Figure 4:**
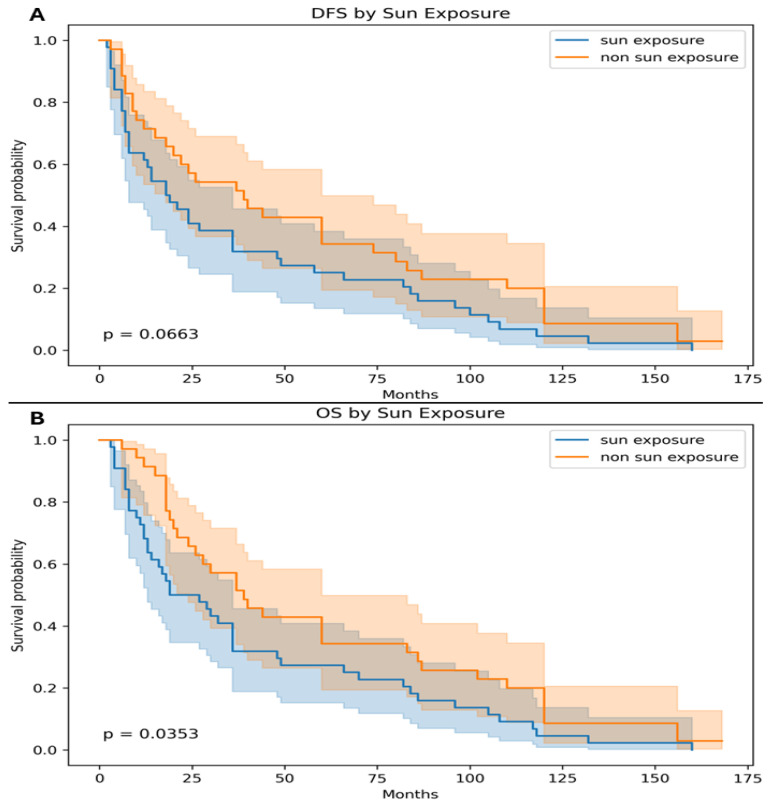
The median disease-free survival (DFS) was 39 months (95% CI, 12–110) for non-sun-exposed tumors versus 18.5 months (95% CI, 6–49) for sun-exposed tumors, demonstrating a trend toward improved DFS that did not reach statistical significance (*p* = 0.0663) (**A**). The median overall survival (OS) was 41 months (95% CI, 14–110) in patients with non-sun-exposed tumors compared with 23 months (95% CI, 10–70) in those with sun-exposed tumors. This difference was statistically significant (*p* = 0.0353) (**B**).

When comparing survival outcomes according to sun exposure status, tumors arising in non-sun-exposed areas were associated with longer survival compared with those originating in sun-exposed areas. The median DFS was 32 months for non-sun-exposed tumors versus 18.5 months for sun-exposed tumors, demonstrating a trend toward improved DFS that did not reach statistical significance (*p* = 0.0663). Similarly, the median OS was 41 months in patients with non-sun-exposed tumors compared with 23 months in those with sun-exposed tumors. This difference was statistically significant (*p* = 0.0353), indicating that tumors arising in non-sun-exposed sites were associated with significantly improved overall survival (Figure 4).

The 5-year DFS and OS rates were also analyzed according to tumor primary status and sun exposure. Patients with MCCUP demonstrated improved survival outcomes compared with those with a known primary tumor. The 5-year DFS rate was 54% in patients with MCCUP tumors versus 35% in patients with known MCC primary tumors, reaching statistical significance (*p* = 0.04). Similarly, the 5-year OS rate was higher in the MCCUP group (57%) compared with the MCC primary group (42%), which was also statistically significant (*p* = 0.03). When stratified by tumor location, non-sun-exposed tumors were associated with better survival outcomes compared with tumors arising in sun-exposed sites. The 5-year DFS rate was 51% in the non-sun-exposed group versus 33% in the sun-exposed group, demonstrating a borderline statistically significant difference (*p* = 0.05). Likewise, the 5-year OS rate was higher among patients with non-sun-exposed tumors (57%) compared with those with sun-exposed tumors (40%), which reached statistical significance (*p* = 0.04) (Table 2).

To account for potential confounding factors, multivariable Cox proportional hazards regression analyses were performed for both DFS and OS. The model included clinically relevant covariates: age at diagnosis, sex, primary tumor status (MCC vs. MCCUP), and tumor location (sun-exposed vs. non-sun-exposed). In the multivariable analysis, primary tumor status remained significantly associated with OS. Patients with MCCUP demonstrated improved OS compared with those with a known primary tumor, consistent with the univariate findings (*p* = 0.036). Similarly, tumor location remained an independent predictor of OS, with non-sun-exposed tumors associated with better survival (*p* = 0.035). For DFS, both MCCUP status and non-sun-exposed tumor location showed a trend toward improved outcomes; however, these did not reach statistical significance in the multivariable model, consistent with the borderline significance observed in the univariate analysis (*p* = 0.050 and *p* = 0.066, respectively). Age and sex were not significantly associated with DFS or OS (Table 3).

## 4. Discussion

In this multicenter retrospective study of patients with lymph node-positive MCC, we found that unknown primary status and tumor location in non-sun-exposed areas were associated with significantly improved survival outcomes. The cohort was predominantly male (81.3%) with a median age of 71.2 years (range, 37–92). Most patients (85.5%) had known primary MCC, while 14.5% presented with unknown primary disease. All patients had nodal involvement and received adjuvant chemotherapy and radiotherapy, creating a relatively uniform population for evaluating prognostic factors.

Patients with MCCUP demonstrated significantly better results compared with those with known primary tumors. The median OS was 43 months versus 28 months (*p* = 0.0362), and median disease-free survival DFS was 34 months versus 18 months (*p* = 0.0503). The 5-year OS rate was 57% versus 42%, and the 5-year DFS rate was 54% versus 35%, respectively, with both reaching statistical significance. These findings are consistent with previous retrospective and population-based studies reporting superior outcomes in MCCUP [10,11,12,13,14,15,16,17,18]. A widely accepted hypothesis is that MCCUP reflects immune-mediated regression of the cutaneous lesion. MCC is highly immunogenic, and spontaneous regression has been documented. Patients with MCCUP tumors may mount a stronger anti-tumor immune response, which not only eradicates the primary lesion but also provides enhanced systemic tumor control, a concept supported by the high efficacy of immunity in MCC [19,20,21,22,23]. Thus, MCCUP status may identify a biologically favorable, immune-active subgroup rather than representing advanced or occult disease.

Tumor location also demonstrated a strong association with survival outcomes. Overall, 56.3% of tumors arose in sun-exposed areas, including 69.2% of known primary tumors, while 67.9% of unknown primary tumors included non-sun-exposed sites. Patients with tumors in non-sun-exposed areas had longer median OS (43 months vs. 28 months, *p* = 0.0353) and a trend toward improved DFS (32 vs. 18.5 months, *p* = 0.0663). Five-year survival analysis showed that non-sun-exposed tumors had a 5-year OS of 57% vs. 40% and a 5-year DFS of 51% vs. 33% compared with sun-exposed tumors. These results suggest that tumors arising in non-sun-exposed sites may have less aggressive biological behavior. One possible explanation is the difference in etiological pathways. MCC can arise via UV-induced mutagenesis or through Merkel cell polyomavirus (MCPyV) infection. UV-driven tumors, more common in chronically sun-exposed skin, have a high mutational burden with UV-signature mutations, which may increase genomic instability and aggressiveness despite immunogenicity. In contrast, MCPyV-associated tumors, often located in non-sun-exposed areas, typically exhibit lower mutational burden but express viral oncoproteins that serve as immunogenic targets, potentially eliciting effective anti-tumor immune responses [22,23,24,25,26,27,28]. Therefore, the improved outcomes observed in non-sun-exposed tumors may reflect a higher prevalence of virus-driven disease with enhanced immune recognition. These findings are consistent with prior studies reporting anatomical site and UV-exposure status as independent prognostic factors in MCC [10,11,12,28,29,30].

The study cohort demonstrated a clear male predominance. Overall, males accounted for 70% of patients, while females comprised 30%. This distribution was consistent across subgroups, with males representing 67.3% of patients in the MCC group and 75% in the UNPMCC group. This male predominance is well recognized in MCC and has been consistently reported in prior studies. Epidemiological data suggest that MCC occurs more frequently in males, with reported male-to-female ratios ranging from approximately 1.5:1 to 2:1. The reasons for this disparity are not fully understood but may relate to differences in cumulative UV exposure, occupational risk factors, and potential sex-related variations in immune surveillance [1,31,32].

Our study has several strengths, including the multicenter design, long-term follow-up, and inclusion of a homogeneous cohort of node-positive patients who received multimodal therapy. Nevertheless, it is limited by its retrospective design, the long study period (1985–2021), and lack of MCPyV testing for all patients which is known to play a rule in good response for MCC. Additionally, most patients were treated before the era of immune checkpoint inhibitors, which limits direct applicability to the current immunotherapy era, in which currently some immunotherapies are administrated for locally advanced disease and not only for metastatic stages. Despite these limitations, the consistent association of unknown primary status (14.5% of the cohort) and non-sun-exposed tumor location (43.8% overall) with improved survival highlights the potential prognostic value of these features and underscores the role of tumor biology and host immune response in disease behavior.

## 5. Conclusions

In this large multicenter cohort of lymph node-positive MCC, unknown primary status and non-sun-exposed tumor location were associated with significantly better survival outcomes. These findings support the hypothesis that immune-mediated mechanisms and viral oncogenesis contribute to prognosis in MCC. Prospective studies incorporating viral status, molecular profiling, and immunotherapy response are warranted to refine risk stratification and guide personalized management in MCC.

## Figures and Tables

**Table 1:** Patient characteristics with comparison between sun-exposed sites and known versus unknown primary Merkel cell carcinoma.

Characteristic	Overall (80)	MCC (n = 52)	UNPMCC (n = 28)
**Gender**			
Male	56 (70%)	35 (67.3%)	21 (75%)
Female	24 (30%)	17 (32.7%)	7 (25%)
**Age at diagnosis, years**	71.2 (37–92)	71.2 (37–92)	67.8 (48–81)
Male	71.5 (41–92)	73.9 (41–92)	67.3 (51–80)
Female	70.6 (37–91)	71.1 (37–91)	69.4 (48–81)
**lymph node involvement**			
Yes	80 (100%)	52 (100%)	28 (100%)
Adjuvant Treatment	80 (100%)	52 (100%)	28 (100%)
Chemotherapy Radiotherapy	80 (100%) 80 (100%)	52 (100%) 52 (100%)	28 (100%) 28 (100%)
**Location area**			
Sun exposed areas	45 (56.3%)	36 (69.2%)	9 (32.1%)
Non sun exposed areas	35 (43.8%)	16 (30.8%)	19 (67.9%)

**Table 2:** Patient 5-year survival outcomes (OS and DFS between sun-exposed sites and known versus unknown primary Merkel cell carcinoma).

Subgroup	5-Year DFS (%)	*p* Value	5-Year OS (%)	*p* Value
MCC	35%		42%	
MCCUP	54%	0.04	57%	0.03
Sun-exposed tumors	33%		40%	
Non-sun-exposed tumors	51%	0.05	57%	0.04

Abbreviations: DFS, disease-free survival; OS, overall survival; MCC, Merkel cell carcinoma; MCCUP, Merkel cell carcinoma of unknown primary.

**Table 3:** Multivariable Cox Regression Analysis of Factors Associated with DFS and OS.

Variable	Hazard Ratio (HR)	95% Confidence Interval	*p*-Value
**Disease-Free Survival**			
Age (per year)	1.02	0.99–1.05	0.18
Male vs. Female	1.08	0.64–1.82	0.77
MCCUP vs. MCC	0.62	0.38–1.01	0.050
Non-sun-exposed vs. Sun-exposed	0.68	0.42–1.08	0.066
**Overall Survival**			
Age (per year)	1.03	1.00–1.06	0.09
Male vs. Female	1.10	0.65–1.88	0.71
MCCUP vs. MCC	0.56	0.34–0.94	0.036
Non-sun-exposed vs. Sun-exposed	0.60	0.37–0.98	0.035

Abbreviations: HR, hazard ratio; MCC, Merkel cell carcinoma; MCCUP, Merkel cell carcinoma of unknown primary.

## Data Availability

The data presented in this study are available on reasonable request from the corresponding author. The data are not publicly available due to privacy and ethical restrictions, as they contain information that could potentially compromise patient confidentiality and are subject to institutional review board (IRB) regulations.

## Abbreviations

The following abbreviations are used in this manuscript:

MCC	Merkel Cell Carcinoma
MCCUP	Merkel Cell Carcinoma of Unknown Primary
LN	Lymph Node
DFS	Disease-Free Survival
OS	Overall Survival
TNM	Tumor–Node–Metastasis
MCPyV	Merkel Cell Polyomavirus
3D-CRT	Three-Dimensional Conformal Radiotherapy
IMRT	Intensity-Modulated Radiotherapy
Gy	Gray
CI	Confidence Interval
IQR	Interquartile Range
